# An Effective Semantic Event Matching System in the Internet of Things (IoT) Environment

**DOI:** 10.3390/s17092014

**Published:** 2017-09-02

**Authors:** Noura Alhakbani, Mohammed Mehedi Hassan, Mourad Ykhlef

**Affiliations:** Information Systems Department, College of Computer and Information Sciences, King Saud University, Riyadh 11543, Saudi Arabia; mmhassan@ksu.edu.sa (M.M.H.); ykhlef@ksu.edu.sa (M.Y.)

**Keywords:** publish/subscribe, semantic, event matching, IoT

## Abstract

IoT sensors use the publish/subscribe model for communication to benefit from its decoupled nature with respect to space, time, and synchronization. Because of the heterogeneity of communicating parties, semantic decoupling is added as a fourth dimension. The added semantic decoupling complicates the matching process and reduces its efficiency. Our proposed algorithm clusters subscriptions and events according to topic and performs the matching process within these clusters, which increases the throughput by reducing the matching time from the range of 16–18 ms to 2–4 ms. Moreover, the accuracy of matching is improved when subscriptions must be fully approximated, as demonstrated by an over 40% increase in F-score results. This work shows the benefit of clustering, as well as the improvement in the matching accuracy and efficiency achieved using this approach.

## 1. Introduction

The IoT is considered to be one of the most important topics in research because of its many beneficial and important applications such as smart cities, smart homes, environmental sensing, and waste management. According to Gartner reports published in 2014 and 2015 [1,2] 4.9 billion connected things will be in use by 2015, and 1.1 billion of these will be used in smart cities. Moreover, this number is predicted to increase to 25 billion by 2020. The rapid increase in the number of communicating sensors has led to an unprecedented scale of data and number of events that need to be communicated as efficiently as possible. Managing such big data using traditional business intelligence or warehousing techniques such as MapReduce or Hadoop is unsuitable for IoT sensors because timely communication based on event processing is required in the IoT. Publish/subscribe event-based middleware can play an important role in this management [3,4,5]. Its communication mode is based on decoupling the interaction between information producers and consumers.

Publish/subscribe event-driven middleware offers a suitable communication paradigm because it supports decoupling in synchronization, time, and space dimensions with limited resource usage, which makes it one of the most suitable modes of communication within the IoT to address many of the challenges faced within this environment. The European Research Cluster on the IoT published a report in March 2015 on the significance of IoT semantic interoperability and its research challenges. The report emphasized the need for semantic publish/subscribe middleware [3]. It further suggested that tight semantic coupling needs to be relaxed or loosened within the publish/subscribe mode of communication to be well suited for the IoT environment. Decoupling means loosening the ties between information publishers and subscribers by removing explicit dependencies between them [6,7,8]. Given the common dimensions of decoupling in the publish/subscribe paradigm, participants can communicate without knowing each other at different times, and neither of them should be blocked from communication [9]. However, most publish/subscribe systems assume syntactic agreement, which is an exact matching of the events and subscriptions between all communicating parties. The communication is tied to a well-defined and agreed-upon schema and content syntax. It is essential that communication among the many heterogeneous data providers and consumers, given their diverse backgrounds, does not require agreement on specific terms beforehand [6,10].

Nonetheless, few studies have been conducted on adding semantic decoupling [6,10] within the publish/subscribe paradigm. This approach faces many challenges such as matching events to the appropriate subscriptions given the absence of an agreement on an event schema or ontology. The other challenge is the efficient delivery of the events given the large number of heterogeneous participants [11]. In addition, the semantic publish/subscribe middleware needs to be scalable. By scalable, we mean that the middleware should be able to accommodate the big data resulting from the large number of sensors and communication events.

Given the need for efficiency in matching publish/subscribe events while recognizing the complexity of matching semantically-related data rather than exact content, we summarize our contribution as follows.

- We propose an architecture that clusters events and subscriptions according to distinct topics and then performs matching within events and subscriptions that fall within intersected clusters.
- We propose an algorithm that matches the events and subscriptions within these clusters and tests the algorithm on a diverse real-world dataset.
- We performed experiments on our algorithm to determine its throughput and accuracy in terms of precision, recall, and F-score.
- We compared our results with the method in [6] and found that our work improved the throughput from 15–18 ms to 1.5–3.0 ms for various ranges of top-K matches.
- We compared our work in terms of the F-score, and, when subscriptions must be fully approximated, our F-score is 0.86 compared to the 0.4 achieved by [6].

The rest of the paper presents related work followed by a motivating scenario for our work and our research questions. Next, we describe the language model we adopted in the publish/subscribe paradigm. Then, we explain the architecture of our proposed system followed by its algorithm. We then present the dataset and experimental results, and finally conclude the paper.

## 2. Literature Review

### 2.1. Publish/Subscribe Paradigm

The publish/subscribe communication paradigm was introduced in the early 90s by Birman and Joseph [12] and, since then, it has proved to be a successful method and widely used in communication within large distributed systems.

Early publish/subscribe systems were implemented over LANs such as TIB/Rendezvous [13] and Elvin [14]. Later, for scalability reasons, they were implemented on WANs such as Gryphon [15] and SIENA [16].

The publish/subscribe paradigm is inherently loosely coupled or decoupled. The communication is performed asynchronously between two parties that are commonly called publishers and subscribers, and alternatively could be called information producers and information consumers.

- Publishers: publish events to the event notification service.
- Subscribers: subscribe to events that meet their desired criteria.
- Event Notification Service: notifies subscribers of published events that match their subscription.

This interaction scheme benefits from decoupling in time, space, and synchronization.

- Time decoupling means that publishers and subscribers do not need to be actively involved at the same time. One of the communicating parties could be disconnected at the time of notification.
- Space decoupling means that interacting parties do not need to know each other or know the number of opposing subscribers or publishers.
- Synchronization decoupling means that publishers do not need to be blocked to publish and subscribers do not need to be blocked to be notified of events.

This inherent communication decoupling nature lends itself to be the main method of communication on the Internet and subsequently within the IoT environment [9].

### 2.2. Publish/Subscribe Paradigm Main Classifications

#### 2.2.1. Topic-Based Publish/Subscribe

The earliest publish/subscribe paradigm falls under the topic-based category. Subscribers register their interest in a certain “topic” and publishers publish their events labeled with a “topic”. The topic-based category is simple and the least expressive of the categories; therefore, its implementation is usually efficient. The most widely used systems that are based on the topic-based publish/subscribe paradigm are SCRIBE [17], ISIS [18], PolderCast [19], and Spidercast [20].

#### 2.2.2. Content-Based Publish/Subscribe Paradigm

Because of the limited expressivity of the topic-based paradigm, another category with added expressivity emerged, called the content-based publish/subscribe paradigm. It is more powerful and can also be used to implement topic-based publish/subscribe algorithms. However, efficient and scalable implementation is harder to achieve given the expressivity and requirements of matching in this paradigm. Some examples of the content-based publish/subscribe paradigm can be found in [21,22,23,24].

The publish/subscribe paradigms presented above are based on exact or range value-matching and none of them consider the possibility that different terminology is used for the same word or concept. Therefore, in a heterogeneous environment where communicating parties do not share the same background, many matches are missed. Thus, a new category of publish/subscribe algorithms has emerged that takes matching semantically-related terms into consideration.

### 2.3. Publish/Subscribe Classes with Semantics and Semantic Coupling

The semantic publish/subscribe paradigm matches subscriptions with events that are related according to their meanings or logic. Thus far, the traditional publish/subscribe paradigm is based on the notion of decoupling or loose coupling in terms of time, space, and synchronization. In contrast, it is tightly coupled with respect to subscriptions, attributes, and crisp operators used to describe the underlying semantics of event exchanges. Traditional publishers and subscribers assume agreement on event types, properties, and values.

- Type coupling happens when the publishers and subscribers agree on the type or class of event instances.
- Property coupling occurs when both participants agree on the set of values for the event attributes or instances.
- Value coupling occurs when both participants agree on the set of values for event attributes or instances.

In the following section, we present the alternative categories of semantic publish/subscribe matching algorithms in the literature.

#### 2.3.1. Concept-Based Methods

Within the concept-based approach, the matching is a Boolean semantic matching based on a concept-level shared agreement or use of domain-specific ontology. S-TOPPS [25] was the first to introduce the concept of semantic matching through the use of a concept hierarchy such as generalization and specialization or mapping functions that allow arbitrary relationships between schema and attribute values for publish/subscribe algorithms. In [26], the authors used a resource description framework (RDF) to represent events rather than the attribute-value pair. They also used graph patterns as subscriptions and a subscription language based on DAML. In [27], the authors used ontologies to understand the semantics of events, then correlated and mapped multiple events using relational operators based on subscriptions. In [28], fuzzy ontology was used, where, again, the events are represented using attribute-value pairs, but the authors add type to their representation.

In [29], a high level ontology library was built which consisted of 20 concepts, then the original data was mapped to a new model using tree structure to enhance efficiency. Ontologies were also used in [30], but they used semantic-based clustering to achieve energy efficient event routing.

Given the small and power limited devices used with the IoT, there are standardized messaging protocols that are lightweight to help achieve energy efficient routing. In [31], the authors mentioned that Extensible Messaging and Presence Protocol (XMPP) is a successful publish/subscribe standard used and [32] presented a comparison between two standardized messaging protocols: Message Queuing Telemetry Transport (MQTT) and Constrained Application Protocol (CoAP). MQTT implements a topic publish/subscribe messaging pattern between subscribers and publishers. All the complexities are implemented in the broker, which is responsible for processing messages organized in a hierarchical topic-based subscription model. CoAP offers the built-in discovery of services and resources, as well as multicast support and asynchronous message exchanges. CoAP also supports a subscribe/notify interaction model, where a server sends a notify message to a client about a change of resource identified by a given URI. Note that a URI corresponds to a topic in the MQTT model [33].

In [34], the authors designed a service-oriented system semantic for publish/subscribe systems. They used an atom-based information container for semantic information that contained the independence of the payload and semantic data, which improved the quality of service for resource-constrained devices. In [35], the authors focused on publish/subscribe systems in the naval ships domain and predicted subscribers. To match them, they mainly used an ontology-based quantitative similarity metric and predicted subscribers based on matching using the semantic similarity between keywords extracted from an event and keywords in historical events. In [36], the authors created complex event services (CES) ontology and defined its formal semantics, then examined their defined ontology in a smart city environment. 

In concept-based publish/subscribe systems, the matching is done using ontologies or a shared agreement of concepts, which hinders terminology decoupling. Furthermore, adding a new term or concept can become cumbersome. Therefore, ontologies are difficult to apply in diverse environments.

#### 2.3.2. Approximate Semantic Event Matching 

Approximate semantic event matching is based on a statistical model built over distributional semantics where the results are ordered according to the approximation. To the best of our knowledge, Liu and Jacobson, who proposed A-TOPPS [37], were the first to introduce the concept of approximate matching through the use of approximate operators rather than crisp operators in publish/subscribe algorithms. They introduced the concept of match degree rather than a crisp Boolean of “Yes” or “No” using a fuzzy set and probability theory to represent approximations, and their results were naturally ordered. Later, major work was presented in [7], where the authors represented the events as an RDF and the subscription as a conjunction of statements that are translated to a pattern to act as a query for events in SPARQL, which is a W3C candidate recommendation query language for RDF. The matching was based on semantically-related events that were then ranked according to similarity; therefore, the result is a real number. Moreover, in [6,8], matching based on semantic relatedness was presented; however, the authors also used the attribute-value model to represent events. A subscription is a sextuple where attributes and values are represented alongside a Boolean to represent whether they are approximated or not and the approximation method to be used. The matching was performed as exact or approximated as required, and the results were ranked according to relatedness. The performance efficiency decreases if the number of attributes that are to be matched to their approximate matches increases. Then, in [38], the authors used the k nearest neighbors (kNN) algorithm to speed up the matching process. The kNN algorithm should be trained manually beforehand for different patterns or types of queries to be matched accurately. 

#### 2.3.3. Thematic Event Processing

In this category, theme tags are added to speed up the matching process. In [8,10], the authors added themes or tags that represent the subscription or event meaning and domain, in addition to their payloads. Hence, the matching is filtered according to matching themes, which increases the throughput. 

From this literature review, we found that event-based middleware using the publish/subscribe paradigm is very suitable for communication between sensors in the IoT because of its inherent nature of decoupling in time, space, and synchronization. Given the advances in IoT, applications such as smart cities, smart homes, and crowd sensing have emerged that operate on a very large scale and communicate using big data. Large-scale communication between heterogeneous parties increases the need for semantic decoupling. Some research has been done to address this need [6,7,8,25,37], but when semantic decoupling is added, the publish/subscribe system becomes much more complex, which negatively affects its efficiency. In our work, we present semantic publish/subscribe middleware with improved efficiency that therefore could be more scalable and well-suited to the nature of the IoT environment.

## 3. Motivation

In a large-scale open environment such as the IoT, messages between different objects need to cross system boundaries; the two main boundaries are syntactic and semantic. The syntactic boundary is concerned with issues such as data formatting and addressing. A semantic boundary appears when different systems use different terminologies for similar or related concepts. Semantic boundaries are inevitable in large and heterogeneous systems.

We can see from the work presented in the literature review, that the topic and content-based publish/subscribe paradigm cross the syntax boundary. Publishers and subscribers agree on specific terms and have predefined large number of rules for the different concepts that are to be used for communication; which means they are tightly coupled by the semantics of the exchanged events. Coupling such agreements is necessary to achieve efficient event transfer within the IoT system. Semantic coupling is defined as “the amount of agreements between participants in the event processing environment on mappings between symbols used in event messages and the meanings they refer to” [8]. Yet, tight semantic coupling within the IoT environment is not realistic since the IoT is highly scalable, open, heterogeneous, and constitutes diverse and non-technical users. So, to enable effective communication within the IoT environment, semantic coupling should be loosened or human agent interference is required. 

To loosen the semantic coupling, concept-based event processing methods were introduced where different parties agree on predefined specific domain ontology or taxonomy. However, updating the ontology to meet changes in the requirements is cumbersome, labor-intensive, and unfeasible due to high levels of heterogeneity at large scales such as smart cities where it can be considered domain agnostic.

In approximate semantic event processing, the matching of events to subscriptions is approximate and not boolean as in concept-based event methods, where it is determined by the predefined ontology. This means that approximate semantic-based methods can deal with uncertainties regarding semantics. Uncertainty, inconsistency, incompleteness, unreliability, or erroneous data is inevitable in IoT, given that communication in IoT is usually between machines or machines and humans that might vary in their reliability level [39]. Therefore, approximate semantic rather than exact boolean is flexible and more suitable to the nature of such communication [6].

Below we propose a sample scenario that can happen in an IoT environment. The scenario presents the need for approximate event matching to a subscription without the need to complicate the subscription.

Suppose Sarah is driving her car in a smart city and looking for a parking spot. In this city, parking garages have sensors that publish their empty parking spots so that nearby interested cars can be notified. Sarah’s car has a sensor that subscribes to events for vacant car parking spaces. A nearby parking garage has three empty spots, and it publishes an event as {parking vacancy = 3}. Sarah’s car sensor subscribes at the same time for {empty parking slot > 0}. Even though the parking garage has a vacant slot, Sarah’s subscription needs would not be met by a traditional event matching system because the choice of wording differs between her subscription and the event published by the garage. To increase the probability of meeting Sarah’s subscription, she could have added alternative synonyms and wordings. This is an added challenge in the IoT environment, where communication is not predefined and all possibilities are very hard to anticipate for every attribute given the scale of such systems. Such environments are naturally large, and the number of communicating entities is huge. Hence, a much more realistic option is to have smarter systems and efficiently match semantically-related events to designated subscriptions.

## 4. Research Questions

Given the current needs of the IoT environment considering its large number of diverse users, IoT middleware should be easy to use by users from heterogeneous backgrounds and should efficiently handle large amounts of data. In this work, we attempt to fulfill these requirements by formulating the following research questions:

Question 1: How can we achieve semantic matching rather than limiting the matching to exact matches using minimal interaction or inputs from users or sensors?

Question 2: How can the system be efficient given the added complexity of loosening the semantic coupling?

The focus of our work is to enhance the publish/subscribe paradigm while maintaining simple and easy-to-use communication. 

## 5. Language Model

### 5.1. Subscription Model

In our proposed system, we define the subscription model as a set of predicates. Each predicate consists of an attribute value model. 

The operator that is used between an attribute and a value is the equality operator (=). 

For example:

$$\begin{array}{cc} \mathrm{Subscription}= & \{{\;\mathrm{Attribute}\;}_{1}={\mathrm{Value}\;}_{1},{\;\mathrm{Attribute}\;}_{2}\;={\mathrm{Value}\;}_{2},\\ & {\;\mathrm{Attribute}\;}_{\ldots }={\mathrm{Value}\;}_{\ldots }\;,{\;\mathrm{Attribute}\;}_{n}={\mathrm{Value}\;}_{n}\;\} \end{array}$$

Formally, a subscription model is defined as the following:

- Let s be a subscription and S be the set of all subscriptions such that s ϵ S
- Each subscription consists of the conjunction of a set of predicates p such that p ∈ P where P is the set of all predicates:
  $$s=\;\left\{\;p_{1}\;\cap \;p_{2}\;\ldots \;\cap p_{n}\;\right\}$$
- Each predicate consists of an attribute value pair (a, v).
- Let a be an attribute and A be the set of all attributes such that a ϵ A.
- Let v be a value and V be the set of all values such that v ϵ V.
  p={(a, v) ϵ (A, V)}

### 5.2. Event Model

The content of an event model is defined as a set of tuples. Each tuple is an attribute value pair. 

For example:

$$\begin{array}{cc} \mathrm{Event}\;=\{{\;\mathrm{Attribute}\;}_{1}= & {\mathrm{Value}\;}_{1},{\;\mathrm{Attribute}\;}_{2}\;={\mathrm{Value}\;}_{2},{\;\mathrm{Attribute}\;}_{\ldots }={\mathrm{Value}\;}_{\ldots }\;,\\ & {\;\mathrm{Attribute}\;}_{n}={\mathrm{Value}\;}_{n}\;\}. \end{array}$$

Formally, an event model is defined as the following:

- Let e be an event and E be the set of all events such that e ϵ E.
- Each event consists of a set of tuples T and each tuple t consists of an attribute value pair (a, v).
- Let a be an attribute and A be the set of all attributes such that a ϵ A.
- Let v be a value and V be the set of all values such that v ϵ V.
  e={t∈T :t=(a, v) ∧ a ϵ A , v ϵ V)}

## 6. Architecture of the Proposed System 

**Figure 1:**
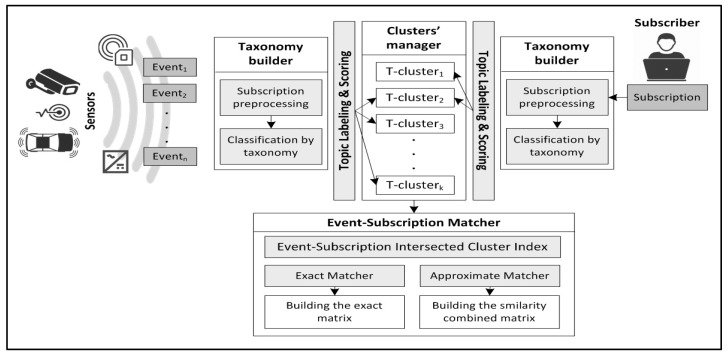
Architecture of the event matching system.

Our system, shown in Figure 1, takes the events and subscriptions as inputs to the taxonomy builder, where it preprocesses the events and subscriptions, then classifies them using taxonomy. Events and subscriptions are labeled for grouping under the corresponding topic clusters. Events that fall within each subscription cluster are matched either exactly or approximately to the subscription predicates as required and a matrix is built for exact and approximate matching accordingly. The resulting matrices are processed, as explained later in Section 7.

## 7. Proposed Algorithm

### 7.1. Explicit Semantic Analysis (ESA) Implementation

In our algorithm, approximate matching is used; we adopt explicit semantic analysis (ESA) [40,41] which has been proven to yield good results for measuring semantic relatedness. Moreover, the corpus used by ESA to calculate similarity is Wikipedia, which is naturally suitable for IoT applications because it is dynamically expanded by users and not limited to a set of predefined ontologies.

We downloaded the Wikipedia dump for 2016. The file is 58 GB and is available at https://dumps.wikimedia.org/enwiki/latest/. We used Apache Lucene [42] to index it.

- From the dump, we built a sparse table where columns correspond to concepts pertaining to an associated article or document d and the rows correspond to the words or terms t that occur in the article.
- Each entry in the table represents the term frequency–inverse document frequency (TFIDF) value of the term $t_{i}$ in the document $d_{j}$.
- To calculate TFIDF, we calculate the following components:
  ○ Term frequency (tf)
    $$\mathrm{tf}\left(t_{i}\;,{\;d}_{j}\right)=\{\begin{array}{cc} \mathrm{count}\;\left(t_{i}\;,{\;d}_{j}\right) & \;\mathrm{if}\;\mathrm{count}\;\left(t_{i}\;,\;d_{j}\right)>0\;\\ 0 & \;\mathrm{otherwise} \end{array}$$
  ○ Document frequency (df) is the number of documents in the collection that contain the term${\;t}_{i}\;$
    $$df_{i}=\;\left|\left\{\mathrm{dk}:t_{i}\;\in {\;d}_{k}\right\}\right|$$
  ○ Inverse document frequency (idf)
    $$(idf_{i}=\log\frac{n}{df_{i}}),$$
    where n is the number of documents in the collection.
- Finally, we apply cosine normalization to each row to counter the differences in document length. $T\left[i,j\right]=\;\frac{T\left[i,j\right]}{\sqrt{\sum_{i=1}^{k}T{\left[i,j\right]}^{2}}}$, where *k* is the number of terms.

Each row in Table 1 is represented as a vector of concepts with their TFIDF values, which in turn expresses the meaning of the term. The TFIDF values reflect the relevance of the concept to the term.

### 7.2. Assigning a Topic: Classification by Taxonomy

To reduce the scale of the search space when finding a relevant match, the events and subscriptions are clustered into disjoint clusters. To do this, we adopted a method inspired by the fact that topic-based publish/subscribe matching systems are much more efficient: we assign a topic to each event or subscription. Events are matched within their relevant topic cluster. The reasoning behind this is that IoT is very diverse in nature, so assigning a topic to subscriptions and events will filter many unrelated events to avoid a time-consuming matching process. We used the AYLIEN text analysis API to classify events and subscriptions according to the IPTC Subject NewsCodes, which originally contained 1400 classes organized into three levels of depth. The dataset used in the experiments was classified into 53 distinct clusters.

### 7.3. Event and Subscription Clustering

**Figure 2:**
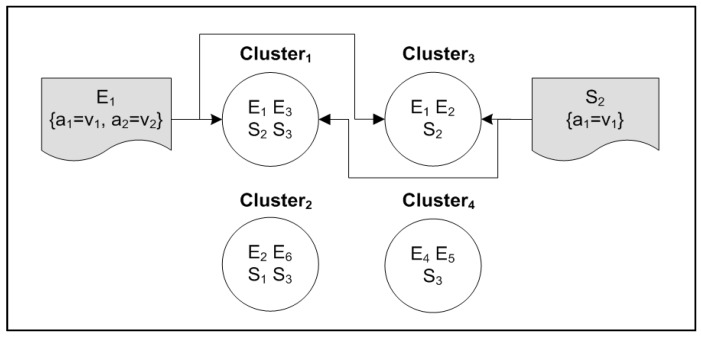
Clustering events and subscriptions.

Each event ID is placed into its attribute and value clusters and, likewise, each subscription ID is placed into its attribute and value clusters. An example to illustrate event and subscription clustering is presented in Figure 2, where subscription 1 ($S_{1}$) falls into clusters {1,2} because ($a_{1}$) and ($v_{1}$) were classified into two different topics. The details of building a cluster list for the events are presented in Algorithm 1. The algorithm takes the set of events as the input and outputs the list of clusters that each event falls into. 

**Algorithm 1** Building the Cluster Index List for Events.**Input:** E: a set of events.**Output:** CE: a list of cluster indexes for events E. 1**Begin** 2 For each event ID (eID) in E do 3 CE←getClusterIndex (eID); 4 End 5return CE; 6**End**

### 7.4. Matching 

Subscriptions are matched to events that fall within the same clusters. Only matching subscriptions to events that fall within the same cluster significantly reduces the matching time and therefore increases the efficiency. Algorithm 2 shows the pseudo code for finding the intersecting clusters of events and subscriptions. The algorithm takes the list of clusters for the events and the ID of a given subscription. 

**Algorithm 2** Finding Event Clusters that Intersect with a Given Subscription.**Input:** CE: a list of cluster indexes for events E.    sID: an ID of a given subscription.**Output:** IC: a list of event cluster indexes that intersect with a given subscription. 1**Begin** 2 CS←getClusterIndex(sID); 3 IC←CE∩CS; 4return IC; 5**End**

### 7.5. Matrix Building 

Subscriptions are matched against events in their respective clusters. Four separate matrices are built for each subscription event pair.

(1) $$\mathrm{Exact}\;\mathrm{Attribute}\;\mathrm{Matrix}\;=\{\begin{array}{ll} 0\; & \mathrm{if}\;(\mathrm{Subscription}-\mathrm{Atttribute}\;\mathrm{requires}\;\mathrm{exact}\;\mathrm{match})\;\mathrm{and}\;(\mathrm{Subscription}-\mathrm{Atttribute})\;\neq \;(\mathrm{Event}-\mathrm{Atttribute})\\ 1\; & \mathrm{otherwise} \end{array}$$

(2) $$\mathrm{Exact}\;\mathrm{Value}\;\mathrm{Matrix}\;=\{\begin{array}{ll} 0\; & \mathrm{if}\;(\mathrm{Subscription}-\mathrm{Value}\;\mathrm{requires}\;\mathrm{exact}\;\mathrm{match})\;\mathrm{and}\;\;(\mathrm{Subscription}-\mathrm{Value})\;\neq \;(\mathrm{Event}-\mathrm{Value})\\ 1\; & \mathrm{otherwise} \end{array}$$

(3) $$\mathrm{Approximate}\;\mathrm{Attribute}\;\mathrm{Matrix}\;=\{\begin{array}{c} \begin{array}{c} \mathrm{ESA}(\mathrm{Subscription}\_\mathrm{Attribute},\mathrm{Event}-\mathrm{Attribute})\\ \mathrm{if}\;(\mathrm{Subscription}\_\mathrm{Attribute}\;\mathrm{to}\;\mathrm{be}\;\mathrm{approximatly}\;\mathrm{matched}) \end{array}\\ 1\;\mathrm{otherwise} \end{array}$$

(4) $$\mathrm{Approximate}\;\mathrm{Value}\;\mathrm{Matrix}\;=\{\begin{array}{c} \begin{array}{c} \mathrm{ESA}(\mathrm{Subscription}-\mathrm{Value},\mathrm{Event}-\mathrm{Event})\\ \;\mathrm{if}\;(\mathrm{Subscription}-\mathrm{Value}\;\mathrm{to}\;\mathrm{be}\;\mathrm{approximatly}\;\mathrm{matched}) \end{array}\\ 1\;\mathrm{otherwise} \end{array}$$

Approximate matrix values can be any value within the range of [0–1]. The numbers in the approximated matrices are based on the ESA result obtained by comparing the attribute or value of the subscription to that of the event whenever a tilde sign (~) is present in the subscription. Approximate matrices are built according to Equations (3) and (4).

All exact and approximate matrices are *I × J*, where *I* is the number of predicates in the subscription and *J* is the number of tuples in the event to be matched. 

The four matrices are combined into one matrix using the entry-wise product, and one conclusive value represents the degree of the match between a subscription predicate and a value tuple. Each predicate-tuple is represented by one match-degree value in the final matrix. Entry-wise product identity (1) and zero (0) elements are obtained from the multiplication operator, which makes it efficient to implement because it can be computed in O (*I × J*).

**Figure 3:**
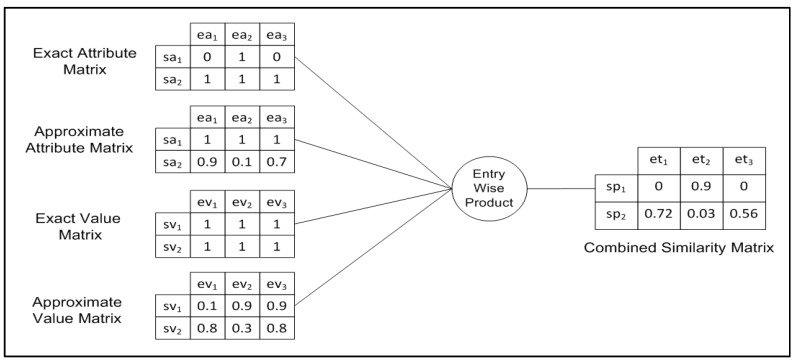
Exact and approximate entry-wise products.

Figure 3 demonstrates building a combined similarity matrix as required by (Algorithm 3). Given a subscription S [] with two predicates and event E [] that has three tuples, we build four 2 × 3 matrices. We use the entry-wise product to generate a single combined similarity matrix that assigns a similarity value for all predicates in the subscription with each tuple in the event.

$$S=\left\{sa_{1}=sv_{1∼},\;sa_{2∼}=sv_{2∼}\right\}$$

$$E=\left\{ea_{1}=ev_{1},\;ea_{2}=ev_{2},\;ea_{3}=ev_{3}\right\}$$

**Algorithm 3** Building the Combined Similarity Matrix.**Input:** IC: a list of event cluster indexes that intersect with a given subscription.    sID: an ID of a given subscription.**Output:** M: a combined similarity matrix. 1**Begin** 2  For each event ID (eID) in IC do 3   M←getSimilarity (eID, sID); 4  End 5return M; 6**End**

### 7.6. Top K Candidates

After obtaining the combined similarity matrix, which maps the similarity between each predicate in the subscription to each tuple in the event, we propose an algorithm to find the best mappings to the required subscription (Algorithm 4). Because we are working with semantic analysis and this can be subjective at times, providing the subscriber with the top-K mappings increases the chance that they will find the required event. 

Algorithm 4 takes the combined similarity matrix as an input and the number of iterations K or number of top events to be returned. After initializing the variables, a priority queue P is created based on the larger sum of values in each column, as calculated in line 5. Because the combined similarity matrix is sorted, the first column has the highest sum, which is the initial vector v. It is added to P and then used as the first element in the $L_{\mathrm{TopK}}$ list. Then, we loop around the combined similarity matrix, as demonstrated in the following example, to find the vector with the highest sum and add it to the priority queue P.

We add the sum of the first column because the combined similarity matrix is sorted and by default it has the highest sum. Then, we start looping and exchange each element from the first column with an element from the next column consecutively within the same row and calculate the sum. This sum is added to the priority queue, which sorts vectors based on the highest sum.

After finding the top-K candidates, the algorithm returns the list of the top vectors $L_{\mathrm{TopK}}$, which corresponds to the best-matching events.

**Algorithm 4** Top-K Candidates.**Input**A: two-dimensional sorted combined similarity matrix K: number of iterations s: summation variable |V|: list of vectors extracted from A**Ouput**$L_{\mathrm{TopK}}$, a list of the top K matching vectors1**Begin**2Initialize A, K,s,|V|
3$L_{A}\leftarrow$RowCountOf (A)4Create P, a priority Queue of vectors from A5s ←
$\sum_{c\in A}c_{i,0}$
6v← the initial vector 7P←
v
8for each *k* ∈ K9    Poll *v* from P10    Add v into $L_{\mathrm{TopK}}$11    for r = 0 to $L_{A}$12     If r > 0 then13       Add $v\left[r-1\right]-1\;\mathrm{into}\;\left|V_{\left[r-1\right]}\right|$14     else15       Add $v\left[r\right]+1\;\mathrm{into}\;\left|V_{\left[r\right]}\right|$16     end if17       P←TopKMapping($A,L_{A},r,\left|V\right|)\;$18    end for19end for20return $L_{\mathrm{TopK}}$21**End**

As a result, Algorithm 1 can build the cluster index list of *n* events in O(*n*) time. Algorithm 2 takes a constant time to find the event clusters that intersect with a given subscription. Algorithm 3 takes a time complexity of O(*n*) to build the combined similarity matrix of a given subscription by searching in specific intersecting clusters. This algorithm improves the search process by searching in specific blocks of events instead of searching all the events, as done in previous methods. Algorithm 4 can find the top-K best mappings within k iterations, and the search space is kept to a minimum and updated with *n* vectors at each iteration. It takes O(*n.m.log*(*m*) *+ n.log*(*n*) *+ k.*$n^{2}$ *+ k.log*(*k*)).

## 8. Dataset

For our experiment, we chose a publicly available dataset [43] that mostly simulates the heterogeneous nature of the IoT environment. Our chosen dataset consists of synthesized events similar to the ones that were produced by the SmartSantander Smart City Project [44] and Linked Energy Intelligence (LEI) [45] dataspace. SmartSantander is a smart city-scale project that consists of 3000 sensors installed in selected European cities in different locations such as bus stops, parking lots, lamp posts, buses, and taxis to monitor different aspects such as traffic, parking availability, and environmental metrics such pollution levels. The LEI dataspace was a smart building project that focused on energy saving and related metrics. 

Table 2 shows some of the parameters that were used to represent the heterogeneous areas in the IoT environment.

### 8.1. Seed Events and Exact Subscriptions

A set of 166 seed events were randomly collected from the datasets. The synthesized events were used as seed events with the exact subscriptions as a match. The ground truth is straightforward for such events and subscriptions. An example of a seed event is:

{type=parking occupied event, Country=Ireland}

### 8.2. Event Set Semantic Expansion

Events were then expanded semantically based on the EuroVoc thesaurus [46]. Selected terms were replaced with their synonym or a related term, which resulted in 14,743 events. Each event consisted of 10 of fewer tuples.

An example of a semantically expanded event obtained from a seed event is:

{scale=none, suburb=galway, continent=europe,

type=vehicle park occupied event, country=eire }.

### 8.3. Approximate Subscription 

An approximate subscription is stated by appending the tilde operator ~ to the attribute and/or value. In the datasets, all attributes and values are approximated. A set of 94 approximate subscriptions were produced by picking selected tuples from the seed events. 

An example of approximate subscription is as follows:

{type∼=parking occupied event∼, country∼=ireland∼} 

### 8.4. Relevance Ground Truth 

In the relevance ground truth, an event matches an approximate subscription if the event contains exact matches with the mentioned tuples or, loosening this criterion, values such that the tuples in events are matched to related or similar predicates in a subscription. The original dataset appended theme tags to subscriptions and events that we discarded because our system only depends on events and subscriptions and does not consider themes.

## 9. Experimental Results 

We evaluated the effectiveness and efficiency of our proposed event matching system to verify it. To test for the efficiency of our proposed service, we used the following metric:

- Throughput measures the number of matched events within a certain time
- To test the effectiveness of our proposed system, we will use the following measures:
- Precision measures the proportion of relevant events discovered by the matcher with respect to all discovered events
- Recall measures the proportion of relevant events discovered by the matcher with respect to all known relevant events from the ground truth
- F-Score combines precision and recall equally such that F1 Score = (2 × Precision × Recall)/(Precision + Recall)

Moreover, we implemented the frontier algorithm proposed in [6] and compared the results with our proposed system.

In the following section, we will present our experimental results, conducted on a Dell Personal Computer that has an Intel Core i7-3770 3.40-GHz CPU with 32 GB of RAM running Java Virtual Machine 1.8 and Microsoft Windows 8 64-bit operating system.

### 9.1. Efficiency: Throughput 

**Figure 4:**
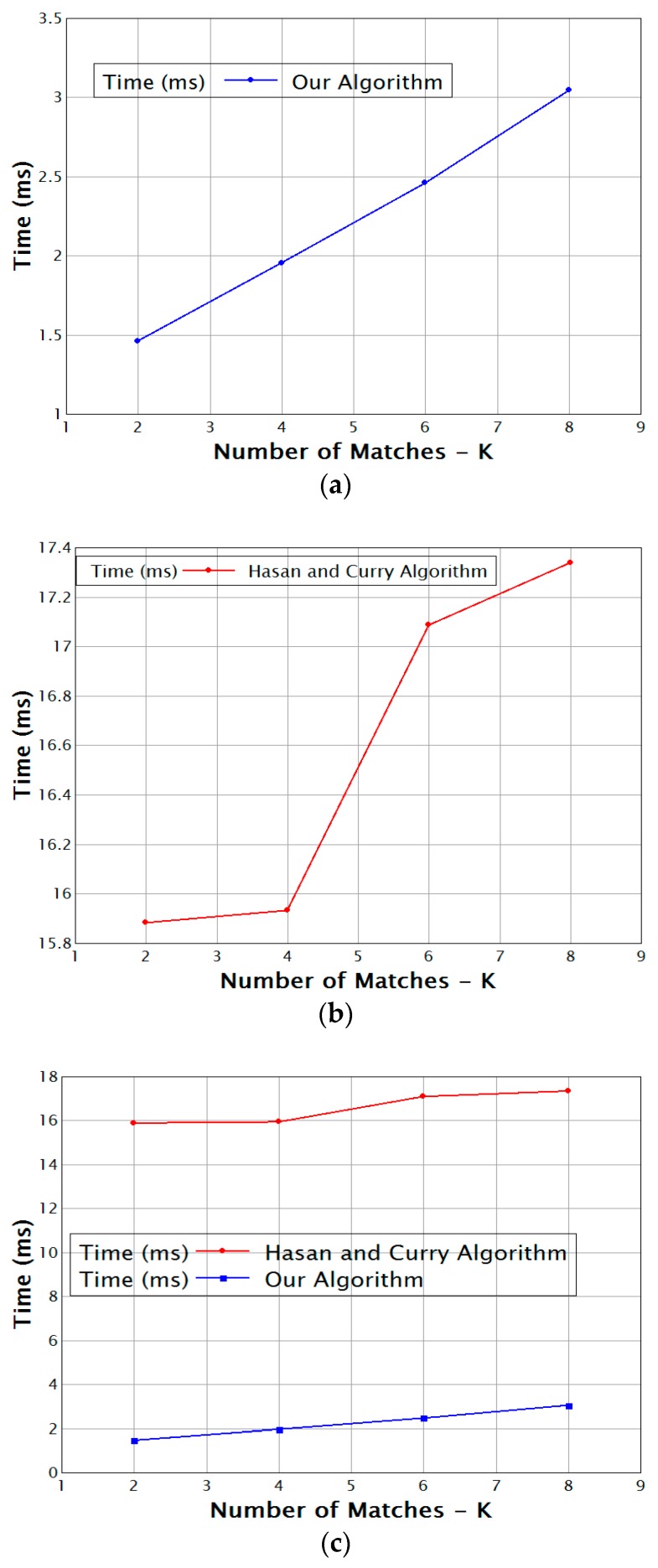
K vs. time (**a**) for our algorithm and (**b**) for the algorithm of Hasan and Curry; (**c**) Comparison of the two methods.

Figure 4 presents the throughput of the event matching system given different numbers of top-k matches (k) for a fixed number of subscription predicates *n* that equals the number of event tuples *m*: *n* = *m* = 10. Our proposed algorithm is almost linear with k and performs much faster than the method in [6] for all values of k. 

**Figure 5:**
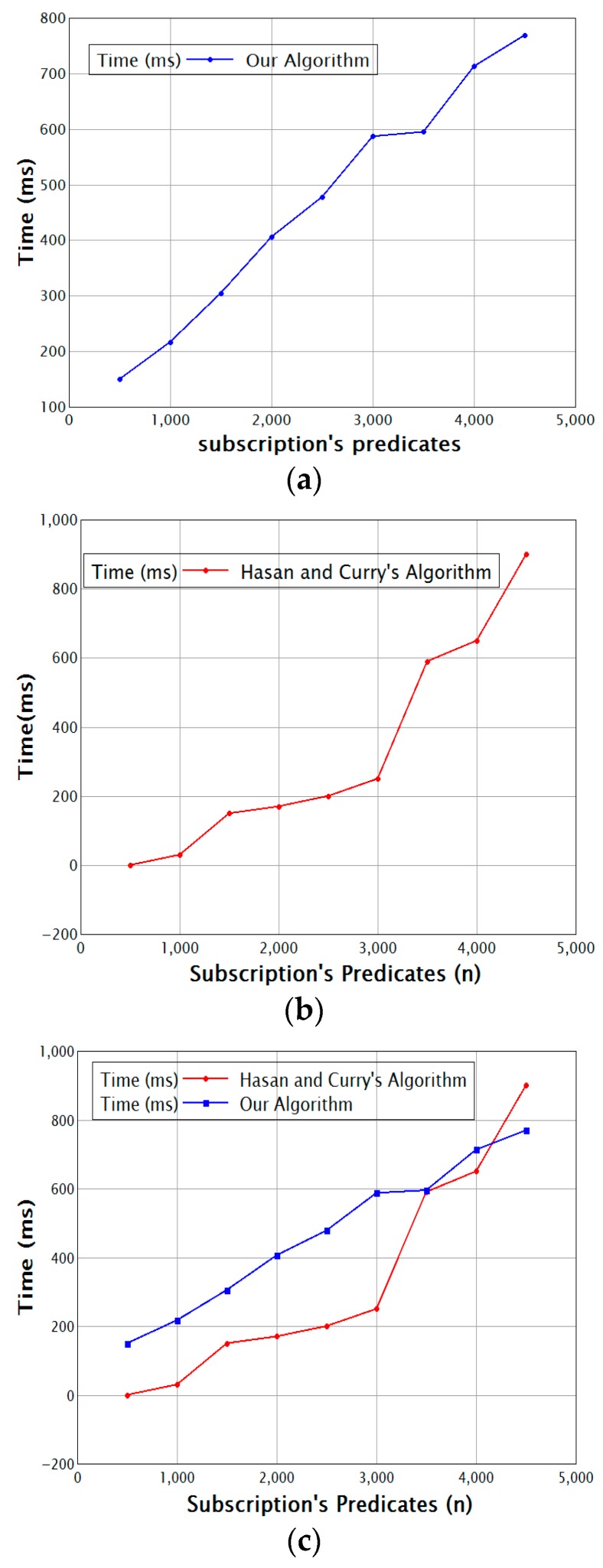
Subscription predicates vs. time (**a**) for our algorithm and (**b**) for the algorithm of Hasan and Curry; (**c**) Comparison of the two methods.

Figure 5 shows the matching throughput for a variable number of subscription predicates. Both graphs show that Hasan and Curry’s algorithm needs less time when the number of predicates is small, but when the number of predicates increases, the throughput in our algorithm increases. The additional time needed grows more slowly, which is due to the clustering, which consumes time for a small number of predicates. However, when the number of predicates increases, the clustering pays off and matching becomes faster.

### 9.2. Effectiveness 

**Figure 6:**
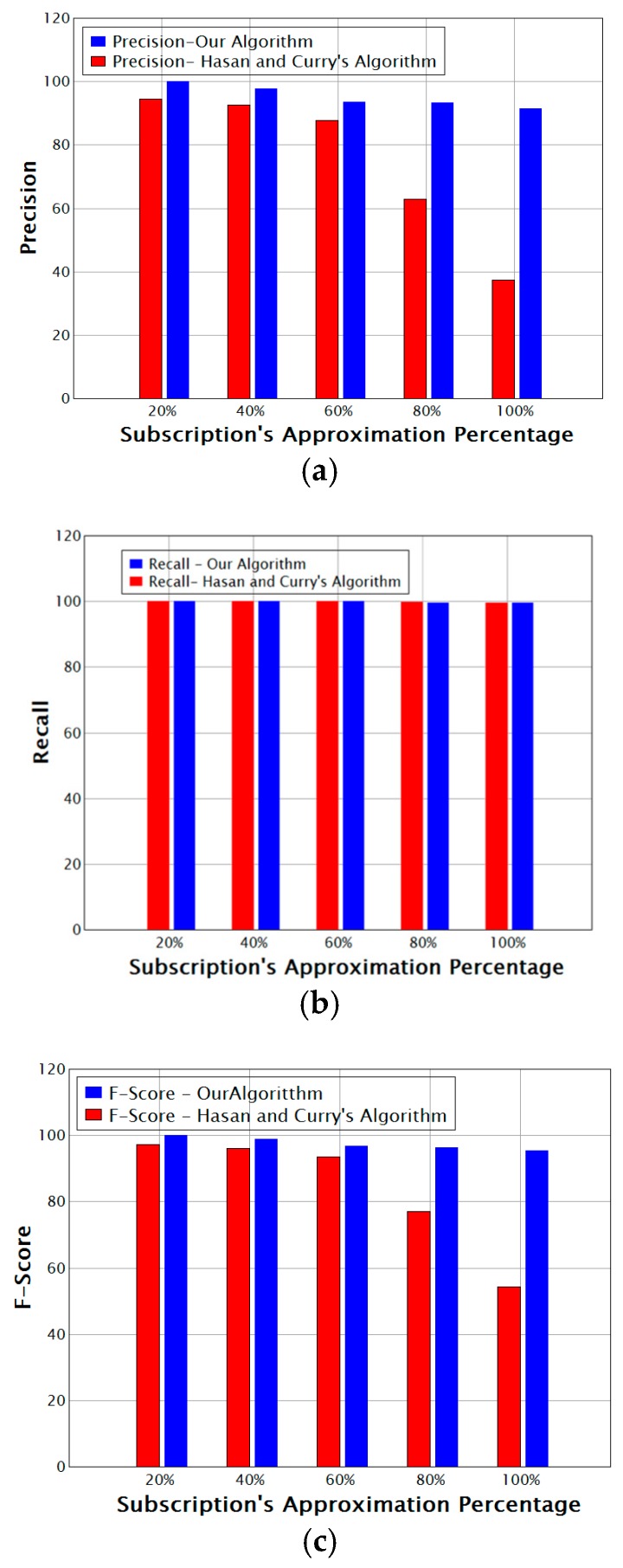
Comparison of Effectiveness Measures for our Algorithm vs. the algorithm of Hassan and Curry; (**a**) Comparison of Precision; (**b**) Comparison of Recall; (**c**) Comparison of F-Score.

Figure 6 shows (a) precision, (b) recall, and (c) F-score versus the percentage of approximated subscriptions for our algorithm compared with that of Hasan and Curry [6]. As we can see in the figure:

- For 80% approximation and above, the precision of the algorithm of Hasan and Curry [6] dropped sharply, whereas our algorithm maintained an ideal precision, decreasing from 93% to 91%.
- The recall value is roughly equal for both algorithms. Yet our algorithm did not consider all events in the matching process.
- Our algorithm achieved better effectiveness with a significant tradeoff between precision and recall that is illustrated by the F-Score; the algorithm of Hasan and Curry [6] was unable to achieve this because of the large number of false-positive matching results.

## 10. Discussion 

Given the nature of IoT, the publish/subscribe model is the most suitable and efficient way of communication. Adding semantic dimension matching to the publish/subscribe model increases the flexibility in communication but complicates matching and reduces efficiency. In this paper, we proposed a method to implement a semantic publish/subscribe model efficiently by grouping diverse events and subscriptions into disjoint clusters classified by topic. Matching is done only between events and subscriptions falling within the intersecting clusters. This clustering saves time needed for the slow matching operation between events and subscriptions that fall into different topics, which are highly unlikely to yield a match. This significantly increases matching effectiveness with respect to precision and the F-Score.

## Figures and Tables

**Table 1:** TFIDF table.

	${\mathrm{Document}}_{1}$	j	${\mathrm{Document}}_{n}$
${\mathrm{Term}}_{1}$	T [$t_{i}\;,\;d_{j}$] = TFIDF		T [${\;t}_{i}\;,{\;d}_{j}$] = TFIDF
i			
${\mathrm{Term}}_{n}$	T [${\;t}_{i}\;,{\;d}_{j}$] = TFIDF		T$\;[{\;t}_{i}\;,{\;d}_{j}$] = TFIDF

**Table 2:** Parameters used.

Air/Wind Parameters	Pollution Parameters	Traffic/Parking Guidance Parameters	Technical Parameters
Wind direction	Particles	Speed	Memory usage
Atmospheric pressure	Solar radiation	Parking	Energy consumption
Wind speed	Ozone		CPU usage
Temperature	Radiation		
Ground temperature	Noise		
	NO_2_		
	CO		
